# Index system of rural human settlement in rural revitalization under the perspective of China

**DOI:** 10.1038/s41598-022-13334-7

**Published:** 2022-06-22

**Authors:** Qi Liu, Decai Gong, Yuxuan Gong

**Affiliations:** 1 University of Science and Technology of China, 96 Jinzhai Road, Hefei, 230026 China; 2 AnHui JianZhu University, 292 Ziyun Road, Hefei, 230601 China; 3 Jiangsu University of Science and Technology, 666 Changhui Road, Zhenjiang, 212100 China

**Keywords:** Ecology, Environmental sciences

## Abstract

Rural revitalization strategies are an important task in China. Currently, it is in the transition from poverty alleviation to rural revitalization. This paper proposes an evaluation index of rural revitalization and development potential based on a summary of previous studies. Together with the TOPSIS method, the corresponding coefficients of each index layer and the weight coefficient of the criterion layer were analyzed. This shows that during the process, the work direction of rural revitalization varies based on different revitalization types. In this study, diagnostic tools are utilized to conduct a potential development analysis of rural human settlements by identifying the main influencing factors for rural revitalization. In addition, an index system for improving rural human settlement strategies is established. Overall, it helps in defining the interventions of reducing and managing the risk of rural vitalization and evaluating the potential ability of rural revitalization. It also suggests that Anhui Province should focus on carrying out the comprehensive revitalization of rural areas according to the different functional positions of the countryside.

## Introduction

Rural area is a complex system composed of social, economic and environmental factors, It is the spatial carrier of working and living^1^. Rural revitalization is rooted in rural development^2^. In international experience and scientific studies^3,4^, Rural development (RD) is a tactic that invented to improve the economic and social life of rural areas.The concept of human settlement is the result of interaction between urban and rural human settlements. Greek scholars^5^ first proposed human settlements as a whole. However, There is a huge difference between urban human settlement and rural human settlement^6^. Population is a main factor of production in in China’s rural development, this is an important foundation to understand the situations in rural China and the implementation of rural revitalization^7^.

China’s urbanization rate rose from 17.92% in 1978 to 60.60% in 2019 (National Bureau of Statistics PRC, 2020).The imbalance between urban and rural regions, as reflected for example in the level of education, the current level of higher education is less than 40% in rural area^8^; In European experience and scientific reviews, A Rural Development Policy (EU) aims to assist rural areas cope with range of environmental, economic and social challenges^9^. Meanwhile, Marsden et al.^10^ explore some conceptual parameters required for the rural development dynamic. Adamowicz present the way of defining rural areas: tactic and development planning in Europe^11^. Some people believe that rural development analysis is a research theme developed in Europe based on scientific reasons and contributions to policy decision-making^12^. Biegańska et al.^13^ focus on changing of demographic and social aspects caused by development around the city in rural areas in Latvia, Poland, and Germany, a number of rural regions face persistent population decline. In Korea experience^14–16^, It discussed rural development policies currently upgraded by the South Korean in terms of questions such as the decrease of agricultural productivity, the depopulation in rural areas. Faradiba et al.^17^ aim to look for the impact of climate, disaster, and social community on rural development. In India experience, Effective policy making for the Rural Development (RD) is the need of hour. RD included reconstruct of life aspects of human that consists of social, political and economic of human beings.

Based on review of rural development index literature, the research on rural index has been covered all over the world. Kaneko et al.^18^ conduct a systematic scoping review to determine the important factors and methodological to be considered in the rural index and provide suggestions for the development of rural index in Japan. Other influential work includes Yokoyama et al.^19^; Abreu et al.^4^ propose the designed rural development index(RDI) that will cover the defining characteristics of regional development. LOBÃO et al.^12^, aim to analyse the extent to which the development level and dynamics of rural regions located in two different countries, Austria and Portugal. Hennebry et al.^20^ analyze the rural development level of Brazilian Amazon municipalities in the decade 2000–2010 based on RDI calculated for more than 400 municipalities. The main objective of Michalek et al.^21^ was to build a comprehensive index to scale the level of rural development and quality of life in rural residents of a specific EU country. The contribution of Liu et al.^22^ is to evaluate the current situation of county level rural areas in the eastern coastal China. The purpose of Kim et al.^23^ is to analyze the current situation and process of rural development in Vietnam and draw lessons for realizing rural sustainable development.

However, all the previously mentioned studies suffer from some serious. Firstly, One criticism of many literature on RDI is theoretical progress, existing studies have identified the concept of RDI, but didn’t figure out the internal composition of RDI and practice of classification at the macro level, specially refer to the human rural settlement. Secondly, in research scale point of view, researchers mainly focus on village scales instead of the national regional level. In the end, The research to date has tended to focus on the different functions of rural development are always seen as relatively independent elements rather than analyzing from an comprehensive and combined perspective. Thus, rural human settlement needs an index system to illustrate the relationship between important elements inside rural revitalization.

This framework was created to provide guidance in the assessment of the potential development ability of rural revitalization. Currently, it is in the transition from poverty alleviation to rural revitalization in China. To smoothly pass through the transition period, a fraction of policies came into existence by the Chinese government, such as the three-year action plan for rural human settlements and the five-year action of rural human settlement improvement, especially rural revitalization strategies^24^. Scholarly and policy work confirms the importance of rural development, and these policies and methods have pointed out the direction for rural development to a certain extent^25^.

In addition, China's No. 1 central document specifically highlights the significance of solving the relationship and problems among three factors, i.e., agriculture, countryside and farmers. The construction of rural revitalization is an important source of farmers’ happiness and sense of achievement Zhang et al.^26^ to completely construct an affluent society. The review of academic sources on the evaluation of rural revitalization is mainly concentrated on the macroscopic level^27^. However, few academic studies have clarified the characteristics of rural human settlements in the microscopic stratification plane. A comprehensive understanding of rural human settlements is considered the foundation and premise condition for the transition from poverty alleviation to rural revitalization in China^28^. The comparison of rural human settlements should include not only multiple regions but also summarize the differences between different regions. It will better reveal the importance of human beings in the process of rural revitalization.

## Research aim

**Figure 1:**
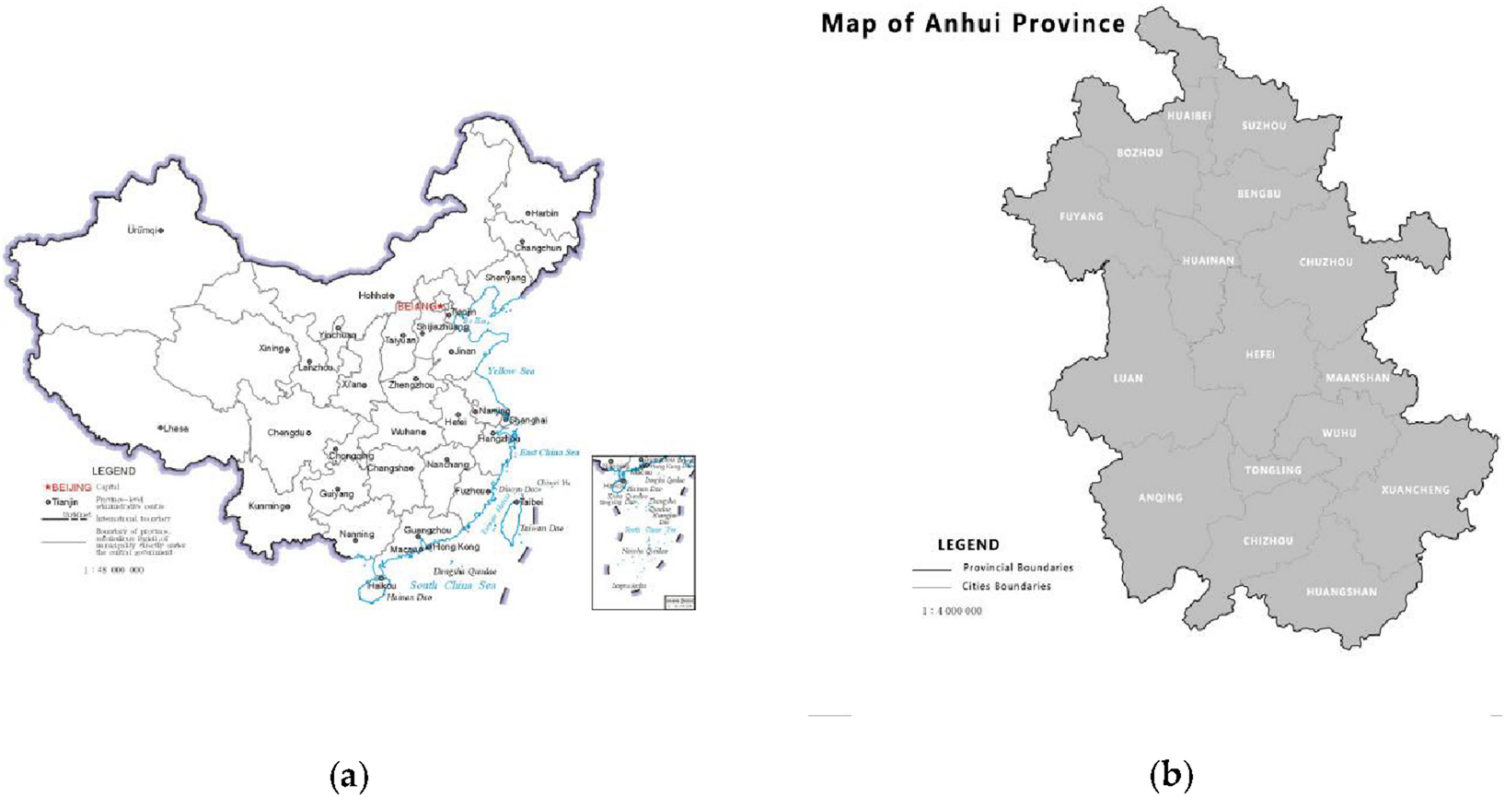
(**a**) The geographical location of China. Source: Obtained from http://o.southgis.com/news/detail/6028; (**b**) The geographical location of Anhui Province. Source:Obtained from http://zrzyt.ah.gov.cn/ztlm/ahsbzdtfw/index.html.

The research objective of this paper is to evaluate the relationship between rural revitalization, i.e., industrial prosperity, ecological livability, rural civilization, effective prosperity, and quality of life in Anhui Province of China (Fig. 1). As the birthplace of China's rural reform and a major agricultural province, in order to better understand the effect of poverty alleviation in Anhui Province and achieve rural revitalization in future research steps. The proposed establishment of an index system is beneficial to evaluate the level of risk of rural revitalization. In addition, it allows us to analyze the potential ability of rural development. From this perspective, this approach is applicable to support scholars and experts in the area of rural revitalization and in the comparison of managing the different levels of development in Anhui Province.

## Theory and background

The major purpose of this study is to estimate the property of human rural settlement .Alongside the historical enhancement of science, there are plenty of indicators to measure development (Gross Domestic Product (GDP); Environmental Sustainability Index (ESI) and Human Development Index (HDI) et al.)^29,30^.

Together with other national development indices(e.g. the EU rural development index, the SRDI, LHDI-low human development index et al.), A number of studies show that the evaluation of development index always combined with theory of sustainability, i.e. the Ecosystem Health Index (EHI) was built up according to the Sustainable Development Goals (SDGs)^31^; Sustainable Rural Development Index(SRDI) is developed on the basis of Rural development index(RDI); Human development index(HDI) is an index that measures key points of human development: healthy of life; education; quality of living. RDI from Kageyamaand^32^ RDI from Abreu^33^ also applied in regional development discussion in Italy^34^ and SRDI in village of Hajij, Iran^35^. Banakar et al. studied that the index was developed can be used to compare with the development level in rural places. Many researchers presented the extent of development in rural areas from different dimensions, i.e. The conceptual Index that proposed with five domains as given below: Economy; Education; Health; Environment; Culture and Leisure, the research comprehensively evaluates the development level of rural comprehensive based on theory of rural regional system from four dimensions: environmental system, resource system, humanistic system and economic system.

Meanwhile, many scholars are also engaged in related research (e.g. the works of Ravallion, Sabina Alkire, James Foster, Kageyama, Abreu et al.). Alkire et al present a new Multidimensional Poverty Index (MPI), which can be used as a method of measuring poverty, Meanwhile, They explained the preponderance, localization, and misreading of multidimensional poverty measurement. Moreover, they examined how rule-based reasoning was used to actual proof application of various indicator in order to make MPI to accord with the SDGs^36–39^. Compared with all those indices, there are also two concrete indicator had established to evaluate RD^32,33^. The indicators that are divided into four parts, but the indicators do have the limitations that endorsed by the literature.

The studies reveal that there are various indices proposed in view of the rural development the research takes into account the literature proposed in developing this conceptual research. Firstly, the indices of RD that are grouped into different dimensions and the indices of SRD Which is from sustainable development point of view, this is a dynamic process instead of a static perspective^40^. Moreover, the main point is to evaluate development process under given index, but it may or may not going in a sustainable way. Secondly, the indices pay almost exclusively attention on national ranking and representation, but does not focus on further development from a specific point of view. In conclusion, This paper not only take dynamic process into consideration (Currently, it is in the transition from poverty alleviation to rural revitalization in China.), but also including the static single indicators measurement to explain how the effect of the first stage of poverty alleviation at present. Thus, both indices (RDI and HDI) together with perspective of china have been considered in the rural human settlement index. There still needs an discussion on how to measure the rural development in a better way, specially in the transition from poverty alleviation to rural revitalization due to special. 

According to the twenty-character policy of rural revitalization, the majority of scholars carried out the evaluation of new rural construction^41^ from different perspectives, such as new farmers, developed agriculture and harmonious rural areas. For the evaluation index of beautiful rural construction, most scholars set up the index system from five aspects: ecological economy, ecological environment, ecological human settlement, ecological culture and ecological support guarantee, which laid a good foundation for the evaluation of rural revitalization. However, there is still a research gap compared with the target requirements of rural revitalization.

In the existing literature, industrial prosperity refers to the change of rural industry from insufficient total amount to structural imbalance^42^ and should be committed to enhancing the comprehensive competitiveness of agriculture^43^. It should also be reflected in agricultural innovation, competitiveness, agricultural product quality and total factor productivity^44^. Industrial prosperity is closely related to comprehensive agricultural production capacity, comprehensive agricultural benefit and product competitiveness. On the other hand, the realization of industrial prosperity is not only related to the new forms of leisure agriculture but also inseparable from the vertical extension of the industrial chain^45^. In the measurement of industrial prosperity, the industrial scale, industrial integration, market demand and farmers' benefit are the standards for township leading cadres to measure industrial prosperity^46^. Additionally, production capacity, unit production level, product quality, production efficiency and versatility can be evaluated. It can be measured by the level of input and output, product quality, industrial competitiveness, green ecological security, and the degree of integration of three industries. Furthermore, industrial prosperity can be constructed from the aspects of agricultural labor productivity, total power of agricultural machinery per capita, commodity rate of agricultural products, employment proportion of the nonagricultural labor force and the proportion of green, science and technology, facilities and ecological agriculture^10,47^. Some scholars measured the level of industrial prosperity from the angles of agricultural production conditions, production efficiency and industrialization level. To reflect the implicit growth of agriculture, the quality and safety of agricultural products are also introduced to jointly measure the degree of industrial prosperity^48^.

In this paper, we selected industrial prosperity, effective governance, ecological livability, rural civilization, and quality of life. These five factors were taken as the main research area in the index system to establish an index system of rural revitalization in human settlement. As a result, efforts have been made to transition from poverty alleviation to rural revitalization in China.

## Materials and method

### Background of index system

The 19th National Congress of the Communist Party of China put forward the "twenty-character" policy of implementing rural revitalization, that is, industrial prosperity, ecological livability, rural civilization, effective governance and good quality of life. In view of the 20-character policy and based on the summary of previous related studies, this research puts forward the evaluation system of rural revitalization and level of development, clarifies the potential of different villages to revitalize and develop, guides the choice of rural development direction, and provides a theoretical basis for the local government to protect rural culture and promote industrial revitalization.

### The measure of index establishment

Based on the current situation, it is in the transition from poverty alleviation to rural revitalization in China. The studies reveal that there are various indices proposed in view of the rural development the research takes into account the literature proposed in developing this conceptual research. But also take both indices (RDI and HDI) together with perspective of china into consideration in sample selection. Thus, sample selection include three parts: According to twenty-character policy of rural revitalization: industrial prosperity, effective governance, ecological livability, rural civilization, and quality of life in China, Academic literature on the topic, Literature review of index selection in china perspective.

The selected evaluation index of rural development level should reflect the development level of villages and towns as comprehensively as possible. According to the principles of scientificity, simplicity, relative independence, objectivity and data availability of index selection, combined with the above research results in China and abroad. Based on the above connotation of "Rural Revitalization", this paper proposes an evaluation index system of "Rural Revitalization" in Anhui Province. According to the design principles of the index system, the five aspects of politics were taken into consideration, i.e., industrial prosperity, ecological livability, rural civilization, effective prosperity, and quality of life. In the specific index system setting, it will be divided into three target levels: the first level is rural revitalization; the second level includes industrial prosperity, ecological livability, rural civilization, effective prosperity, and quality of life; and the third level is composed of several single evaluation indicators under the second level, with a total of 13 indicators (Table 1) as the third level scalar layer.

**Table 1:** Index establishment of Rural Revitalization.

Target layer	Criteria layer	Indicator layer
Rural revitalization	Industrial prosperity^4,5,7,13,17^	Productive capability
		Planting area of main crops
		Output rate of rural labor force
	Ecologically livable^11,18,19,21^	The level of Rural health
		Rural tap water penetration rate
	Rural civilization^22–25^	Education level of farmers
		Number of community service facilities
	Effective governance^27,28,30,31^	Coverage rate of cultural relics
		Household biogas utilization rate in rural areas
	Quality of life^35,36,40,42^	Per capita disposable income of rural residents
		Consumption level of rural residents
		Rural employment rate
		Penetration rate of rural electricity consumption

The following evaluation indicator system was developed:

### Data source

Anhui Province and 2019 were selected as the research unit and time. The data are constructed by spatial and attribute data separately. The spatial boundary included administrative boundaries (the 1:1000000 basic geographic information database of China). Attribute one comes from statistical data collected by the related state departments. The data included, but were not limited, infrastructure data, environmental data, and social and economic data. These data came from the China Urban Rural Construction Statistical Yearbook, China Rural Poverty Monitoring Report, China Statistical Yearbook, China Social Statistics Yearbook, China Environmental Statistics Yearbook and China Rural Statistical Yearbook.

### Methods

In the selection of method stage,the studies shows that during the preliminary decision-making phases, there are various methods. i.e. SWOT^49^; Delphi^50^ et al. i.e. On account of the SWOT analysis and the attitudes of the local population, that determine the SRD index which comprise twenty indicators^51^; Abreu et al.^4^ propose the SRD index through a Delphi method.Which consist of 25 indicators from demographic, economic, environmental, and social welfare aspect.

However, how to select a relevant MADM which is Multiple Attribute Decision Making method for a given question has been highlighted in studies^52^. It is necessary to do the comparison for method^53^. Firstly, in comparison to the subjective fixed weight methods for instance the Delphi method, comprehensive index method, fuzzy comprehensive evaluation method, the analytic hierarchy process method (AHP) etc., the entropy weighting method can explain the results acquired more exact and objective. It conclude three steps: set up the assessment matrix, rescale the matrix and calculate the entropy weight.

Secondly, Yoon et al. had developed TOPSIS, it’s definition is Technique for Order Preference by Similarity to Ideal Solution^54^, and it is also an MCDM(multi-criteria decision making). TOPSIS is derived from the idea that the geometric distance of chosen one should be the shortest from the positive ideal solution and the longest from the negative ideal answer, which is the method to optimize responses^55^, Based on the available literature studies, specially in contrast to the sing response optimization techniques^56^, it is clear that the benefits of the TOPSIS model is more simple, reasonable, understandable, high computing efficiency. Therefore, and in the existing methodologies, researchers can test alternatives and decision criteria through the use of numerical tool.

Thus, according to the summary of previous related studies, this paper puts forward the evaluation index of rural revitalization and development potential and analyzes the corresponding coefficients of each index layer and the weight coefficient of the criterion layer. The TOPSIS method was selected by comparison with the comprehensive index method, fuzzy comprehensive evaluation method and AHP method.

#### Building decision matrix

1 $${\text{x}} = \left\{ {\begin{array}{*{20}c} {{\text{x}}_{11 } {\text{x}}_{12} \cdots {\text{x}}_{1,13} } \\ {{\text{x}}_{21} {\text{x}}_{22} \cdots {\text{x}}_{2,13} } \\ \vdots \\ {{\text{x}}_{16,1} x_{16,2} \cdots {\text{x}}_{16,13} } \\ \end{array} } \right\}$$

#### Dimensionless method

Different dimensions were observed in the evaluation index of rural revitalization of Anhui Province. For the convenience of data processing, dimensionless data were carried out first, which means that all units of the equation of a physical quantity are removed by replacement with a suitable variation to simplify the purpose of calculation. In this paper, the method of extreme difference transformation is used. In the decision matrix:

2 $${\text{x}} = \left( {{\text{x}}_{{{\text{ij}}}} } \right)_{16 \times 13}$$

Target j for i cities, then:

3 $$z_{ij} = \frac{{x_{{ij - \mathop {\min }\limits_{i} x_{ij} }} }}{{\mathop {\max }\limits_{i} x_{ij} - \mathop {\min }\limits_{i} x_{ij} }},\;\;j = 1,2,3 \ldots 13$$

Therefore, turning to the indicator, at the same time 0 ≤|x|≤ 1, the matrix will be the new matrix:

4 $${\text{z}} = \left( {{\text{z}}_{{{\text{ij}}}} } \right)_{16 \times 13}$$

#### Indicator wet set

To reflect the difference between the main and secondary evaluation factors and increase the comparability, the entropy weight method is used to determine the weight of each evaluation index. The entropy weight method can determine the index weight according to the amount of information contained in the objective index. It helps to avoid the interference of index information data acquisition and has high reliability. The steps are as following, For matrix:

5 $${\text{z}} = \left( {{\text{z}}_{{{\text{ij}}}} } \right)_{16 \times 13}$$

To calculate the average value of every evaluating indicator:

6 $$\overline{{{\text{z}}_{{\text{j}}} }} = \frac{1}{16}\mathop \sum \limits_{{{\text{i}} = 1}}^{1} {\text{z}}_{{{\text{ij}}}} (\begin{array}{*{20}c} {j = 1,2,3 \ldots 13)} \\ \end{array}$$

Then, the standard deviation of the evaluation value is calculated:

7 $${\text{t}}_{{\text{j}}} = \sqrt {\frac{1}{16 - 1}\mathop \sum \limits_{{{\text{i}} = 1}}^{16} \left( {{\text{z}}_{{{\text{ij}}}} - {\overline{\text{z}}}_{{\text{j}}} } \right)^{2} } (\begin{array}{*{20}c} {j = 1,2,3 \ldots 13)} \\ \end{array}$$

Calculate the coefficient of variation of the evaluation value of each index

8 $$M_{j} = \frac{{t_{j} }}{{\overline{z}_{j} }}(\begin{array}{*{20}c} {j = 1,2,3 \ldots 13)} \\ \end{array}$$

Finally, the index weight is obtained by normalization:

9 $$w_{j} = \frac{{M_{j} }}{{\mathop \sum \nolimits_{j = 1}^{13} M_{j} }}(\begin{array}{*{20}c} {j = 1,2,3 \ldots 13)} \\ \end{array}$$

#### Comprehensive ranking of indicators

First, the weighted standardized matrix is calculated from the normalized decision matrix and the index weight vector to obtain the weighted standardized moment array. Second, to determine the positive and negative theoretical solutions, positive and negative theoretical solutions can be obtained from the previous step. Third, calculate the distance from the evaluation vector to the positive and negative ideal solutions every year. The distance value from the evaluation vector to the positive and negative ideal solutions is $L_{i}^{ + }$ and $L_{i}^{ - }$:

10 $$L_{i}^{ + } = \sqrt {\mathop \sum \limits_{j = 1}^{13} \left( {N_{ij} - N_{j}^{ + } } \right)^{2} } (\begin{array}{*{20}c} {j = 1,2,3 \ldots 13)} \\ \end{array}$$

Finally, the relative closeness of the evaluation vector every year is calculated:

11 $$K_{i}^{*} = \frac{{L_{i}^{ - } }}{{L_{i}^{ - } + L_{i}^{ + } }}(\begin{array}{*{20}c} {j = 1,2,3 \ldots 13)} \\ \end{array}$$

## Results and discussion

### Visualization chart

**Figure 2:**
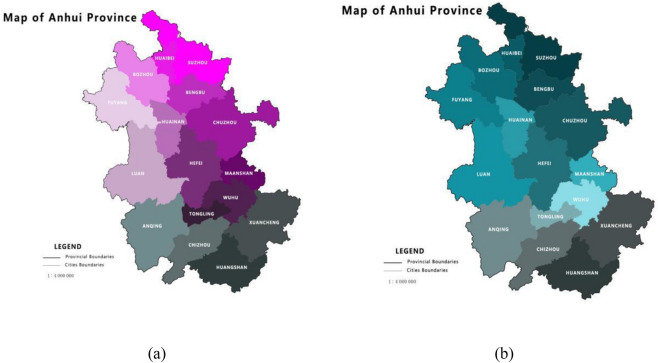
(**a**) The overall rural revitalization index level of Anhui Province in 2019; (**b**) The level of industrial prosperity in Anhui Province.

The research aim of this paper focused on evaluating the relationship between rural vitalization, i.e., industrial prosperity, ecological livability, rural civilization, effective prosperity, and quality of life in Anhui Province of China (Table 2). *In Anhui province, there are 16 cities totally and Hefei is the capital city of the province.* After data processing (Fig. 2), different colors represent different results in numerical order. The lighter the color is, the higher the ranking. Similar to the value of $k^{ + }$, according to the comprehensive evaluation value, the larger the value is, the better the effects of rural revitalization. From a geographical point of view, the score of the northwest is higher than that of the southeast area. As shown below, to build the rural revitalization demonstration area, it should focus on areas with higher development levels in the early stage (Table 3).

**Table 2:** The weight of the evaluation index.

Area	Information entropy	Information utility value	Weight coefficient
Productive capability	0.8905	0.1095	7.60%
Planting area of main crops	0.8897	0.1103	7.65%
Output rate of rural labor force	0.9262	0.0738	5.12%
The level of Rural health	0.9062	0.0938	6.51%
Rural tap water penetration rate	0.9328	0.0672	4.66%
Education level of farmers	0.8186	0.1814	12.59%
Number of community service facilities	0.8894	0.1103	7.67%
Coverage rate of cultural relics	0.7901	0.2099	14.56%
Household biogas utilization rate in rural areas	0.8738	0.1262	8.76%
Per capita disposable income of rural residents	0.8275	0.1725	11.97%
Consumption level of rural residents	0.8924	0.1076	7.46%
Rural employment rate	0.9565	0.0435	3.02%
Penetration rate of rural electricity consumption	0.9650	0.0350	2.43%

**Table 3:** Evaluation results in Anhui province(cities).

Area	${L}_{i}^{+}$	${L}_{i}^{-}$	$K^{ + }$	Ranking
Hefei	0.733	0.518	0.414	3
Huaibei	1.010	0.113	0.101	16
Bozhou	0.786	0.498	0.388	4
Suzhou	0.848	0.457	0.350	6
Bengbu	0.788	0.370	0.319	10
Fuyang	0.753	0.730	0.492	2
Huainan	0.807	0.374	0.317	12
Chuzhou	0.851	0.411	0.326	8
Luan	0.845	0.394	0.318	11
Maanshan	0.922	0.407	0.306	13
Wuhu	0.809	0.451	0.358	5
Xuancheng	0.831	0.334	0.287	14
Tongling	0.979	0.270	0.216	15
Chizhou	0.878	0.414	0.320	9
Anqing	0.747	0.388	0.342	7
Huangshan	0.710	0.701	0.497	1

### Development level of index

**Figure 3:**
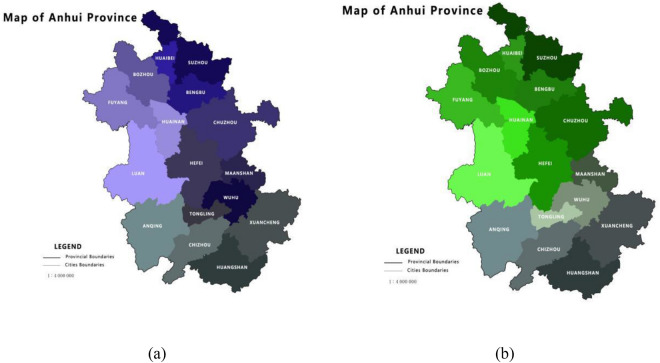
(**a**) The level of ecological liveability in Anhui Province; (**b**) The level of rural civilization in Anhui Province.

**Figure 4:**
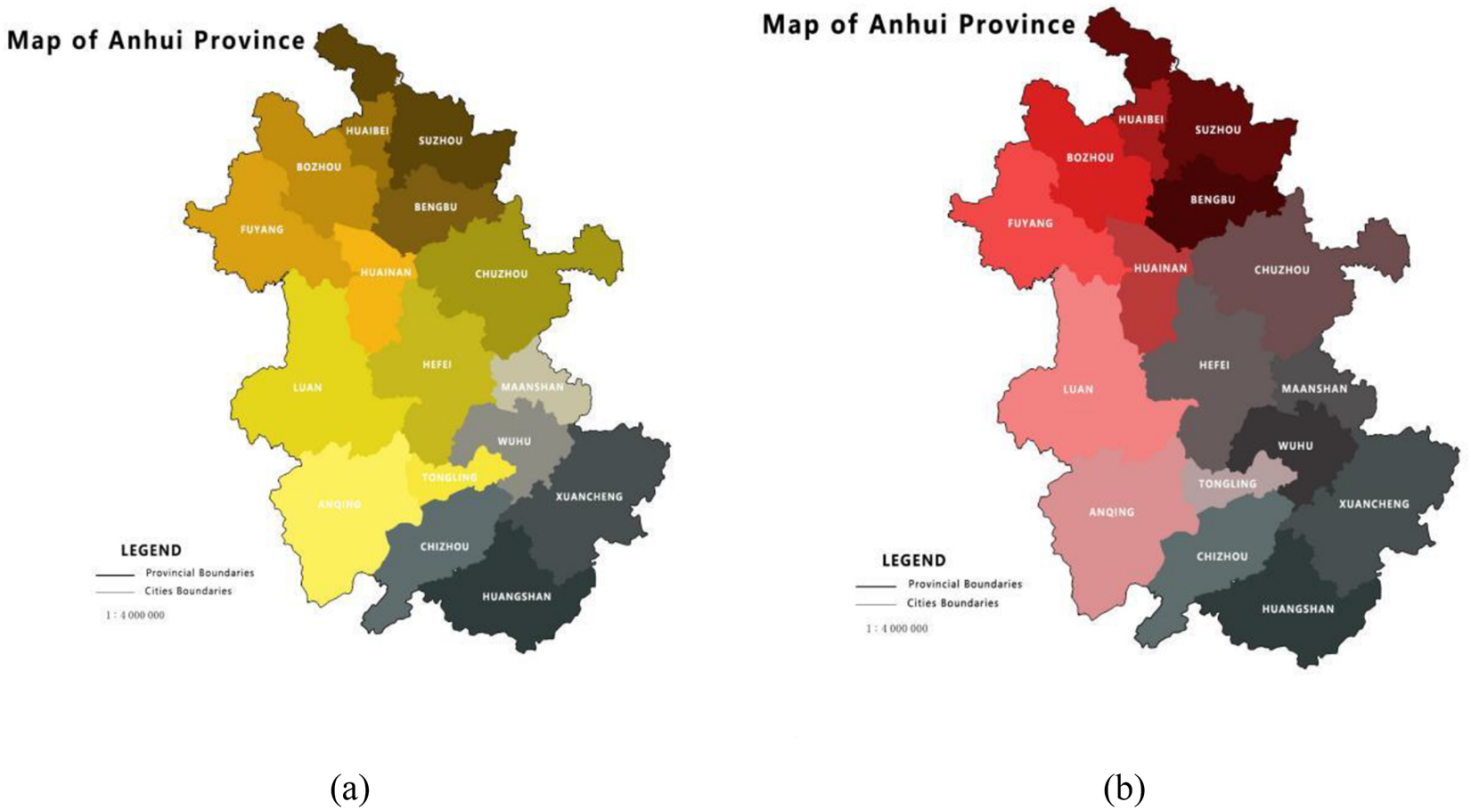
(**a**) The level of effective governance in Anhui Province; (**b**) The level of quality of life in Anhui Province.

To reflect Anhui Province more objectively and clearly, the criteria layers should also be evaluated separately (Fig. 3). Table 4 shows the ranking in five different criteria layers. Huangshan plays a significant role in effective governance (Fig. 4), which leads to its high comprehensive ranking. Hefei, as a provincial capital place in Anhui, has the highest level of quality life, but compared to other aspects, it needs to be improved through effective governance and rural civilization.

**Table 4:** Ranking results in five aspects of Anhui province.

Area	Industrial prosperity	Ecologically livable	Rural civilization	Effective governance	Quality of life
Hefei	4	6	6	8	1
Huaibei	16	16	15	16	16
Bozhou	5	3	2	10	11
Suzhou	2	2	3	15	13
Bengbu	6	14	4	12	5
Fuyang	1	1	1	6	4
Huainan	8	15	10	3	8
Chuzhou	3	11	7	11	10
Luan	7	4	8	9	9
Maanshan	15	10	12	14	3
Wuhu	10	7	11	7	2
Xuancheng	11	8	13	5	6
Tongling	12	13	14	13	14
Chizhou	14	12	16	2	15
Anqing	9	5	5	4	12
Huangshan	13	9	9	1	7

Furthermore, solving the problem of differentiation in rural areas, applying contemporary types and classifications, and putting personalized methods into practice will certainly contribute to the development of rural revitalization^2^.

From a multidimensional perspective, rural revitalization is a strategy to improve rural resilience, and it is also useful to promote sustainable development^57^. Different rural areas play different roles in the end. Eco-friendly villages pay attention to solving the contradiction between ecological environment protection and traditional ecological poverty alleviation and construct the realization path of green poverty reduction^58^; deep poverty-stricken areas eliminate poverty by developing industries with regional characteristics, implementing ecological protection and green development, relocation and other measures^59^. On this basis, to provide financial security and enhance the endogenous development capacity of developing areas^60^, the goal is to help farmers solve the problem of financing for development and constantly innovative financial services mechanisms^61^.

Consequently, before rural revitalization, it is necessary to carry out an evaluation of the situation of poverty alleviation and the development potential of rural revitalization in various regions to select countermeasures and carry out the work.

## Conclusion

Based on the assessment of the current poverty alleviation and the preliminary judgment of the existing data using the TOPSIS method, the following conclusions are drawn:

To achieve rural revitalization, it is necessary to consider the connection between poverty alleviation and rural revitalization. Currently, rural revitalization is the goal of rural development, poverty alleviation is the foundation and premise of rural revitalization, and rural revitalization is the consolidation and deepening of poverty alleviation in China. The relationship between poverty alleviation and rural revitalization is the integration of content, interaction and consistency of the main body. To overcome the relationship between the stage of poverty and solving long-term relative poverty should be the first step, and then it can help in solving the problem of extreme poverty. Then, it will be possible to achieve rural revitalization^62^ after overcoming poverty and the key points. Thus, China's No. 1 central document pointed out that give full play to the rural industry supply, ecological barriers, cultural heritage and other functions to consolidate and expand the achievements of poverty alleviation and rural revitalization effective convergence in 2021.

To achieve rural revitalization, it is necessary to give full play to the rural industry supply, ecological barriers, cultural heritage and other functions. The ecological environment is the greatest advantage and wealth in rural areas. Making full use of good ecological resources and achieving rural ecological livability is the key to effectively linking rural revitalization. Rural cultural revitalization can promote the development of the rural cultural industry and is an open channel for local tourism, folk customs and intangible cultural heritage. Furthermore, rural cultural protection not only protects the characteristic culture but also promotes local economic development. Industrial development compacts the material foundation from poverty alleviation to revitalization, explores the development direction of rural industry and seeks industrial revitalization.

To achieve rural revitalization, it is necessary to achieve regional classification and to excavate endogenous potential, which is critical to rural regions. Rural classification is applied in many countries. Marsden1995 analyzed the development levels of agricultural systems and divided these villages into different types: production-oriented villages and integrative villages according to the period of rural development. To collect rural migration models, Bijker et al.^63^ evaluated the popularity degree of rural areas according to the landscape and employment and divided rural areas into popular and less popular areas. Thus, it is important to start a diagnostic system to classify various villages. Thus, the symbol to measure whether rural revitalization occurs mainly lies in whether the special and necessary functions of the areas have been fully played. Anhui Province has different rural resource endowments, various industrial types and different stages of rural historical development. In general, the differentiation of rural functions is an indisputable fact. The main direction of development at different stages should also be different.

Currently, it is in the transition from poverty alleviation to rural revitalization in China. Achieving rural revitalization is a long-term dynamic construction process. On the basis of the achievements of poverty alleviation, evaluating the effectiveness of the work is necessary and urgent. Therefore, the next stage should focus on how to carry out the comprehensive revitalization of the rural area according to the different functional positioning of the countryside.
